# A Case of Acute Eosinophilic Leukemia with a Novel *PHF*6 Mutation

**DOI:** 10.1155/2021/5574766

**Published:** 2021-07-03

**Authors:** J. J. Lipof, E. J. Huselton, C. S. Zent, A. Evans, B. Zhang, P. G. Rothberg, J. M. Bennett

**Affiliations:** ^1^Division of Hematology/Oncology, University of Rochester Medical Center, James P. Wilmot Cancer Institute, Rochester, NY, USA; ^2^Department of Pathology and Laboratory Medicine, University of Rochester Medical Center, Rochester, NY, USA

## Abstract

Acute eosinophilic leukemia (AEL) is a rare form of acute myeloid leukemia (AML) that requires prompt exclusion of reactive etiologies of eosinophilia and identification of an underlying acute myeloid neoplasm. Myeloid neoplasms with prominent eosinophilia often have rearrangements in the platelet-derived growth factor receptor *α* (*PDGFRA*) or *β* (*PDGFRB)* or are associated with core-binding factor AML. In this report, we describe a 35-year-old male presenting with chest discomfort and altered mental status, found to have marked leukocytosis with eosinophilic predominance and an elevated blast count. Bone marrow aspirate and biopsy findings were morphologically consistent with AEL. Fluorescence in situ hybridization (FISH) and standard karyotype analysis did not reveal any abnormalities, and mutation analysis using next generation sequencing (NGS) revealed a pathogenic mutation in *PHF*6. Cardiac work-up revealed findings suggestive of eosinophilic myocarditis. High-dose glucocorticoid therapy was initiated followed by standard intensive induction chemotherapy with cytarabine and idarubicin. He experienced a rapid reduction in peripheral blood eosinophil and blast count and was found to be in a complete remission at the time of his postinduction bone marrow examination. He underwent allogeneic stem cell transplantation with a matched sibling donor after consolidative high-dose cytarabine and remains in remission at the time of this report, 6 months following his initial diagnosis. The rarity of this condition has resulted in a paucity of data to guide management. Additional studies are needed to better characterize this entity and inform optimal management strategies to attain a long-term sustained remission in these patients.

## 1. Introduction

The differential diagnosis of peripheral blood eosinophilia is broad and includes both malignant and reactive etiologies. Triggers for reactive eosinophilia include drugs, infections (commonly parasitic), and allergies. Patients are diagnosed with hypereosinophilic syndrome (HES) when there is persistent eosinophilia (>1.5 × 10^9^/L) for greater than 6 months, absence of a clear cause, and clinical manifestations with evidence of organ involvement [1]. Most of these cases are determined to be reactive, resulting from cytokines released in the setting of a known trigger. Rarely, a clonal aberration is identified, and the eosinophilia is found to be related to a myeloproliferative neoplasm (MPN) such as chronic eosinophilic leukemia (CEL), acute myeloid leukemia (AML), or acute lymphoblastic leukemia (ALL). Here, we present a case of acute myeloid leukemia with minimal differentiation and prominent eosinophilia, with a pathogenic mutation involving plant homeodomain finger 6 (*PHF*6) with a variant allele frequency (VAF) of 13%, as well as a mutation in *ZRSR*2 (VAF: 73%).

## 2. Case Presentation

**Figure 1:**
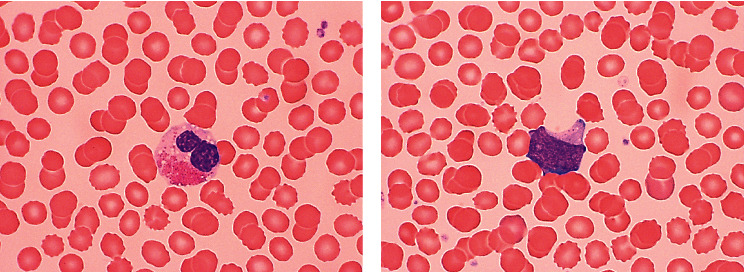
Peripheral blood smear: (a) mature eosinophil with hypogranulation and prominent vacuoles and (b) a blast with rare azurophil granules. Wright–Giemsa stain: 1000x.

A 35-year-old male with no significant past medical history presented to an outside hospital with fever to 38.9 C, altered mental status, headache, and chest pain. EKG showed normal sinus rhythm with diffuse ST segment depression in leads II, aVF, and V3–V6. He was found to have leukocytosis with a white blood cell (WBC) count of 55.45 × 10^9^/L (normal range: 4.2 to 9.1 × 10^9^/L), thrombocytopenia with a platelet count of 102 × 10^9^/L (150 to 330 × 10^9^/L), and hemoglobin of 15.7 g/dL (13.7 to 17.5 g/dL). The leukocyte differential count revealed marked eosinophilia with an absolute eosinophil count of 16.64 × 10^9^/L (0.0 to 0.5 × 10^9^/L), representing 30% of the total WBC count. The remainder of the differential was significant for 44% neutrophils, 5% lymphocytes, 4% monocytes, 2% basophils, and 13% blasts (Figure 1). A transthoracic echocardiogram showed a low normal systolic function with an estimated left ventricular ejection fraction of approximately 55% and concern for a left ventricular (LV) apical thrombus versus endomyocardial fibrosis. He subsequently underwent cardiac magnetic resonance imaging (MRI) which revealed large areas of subendocardial and midmyocardial delayed enhancement most prominent in the LV apex, suggestive of eosinophilic myocarditis, and confirmed the LV apical thrombus. A left heart catheterization revealed no evidence of coronary artery disease. He was transferred to our institution for further care.

**Figure 2:**
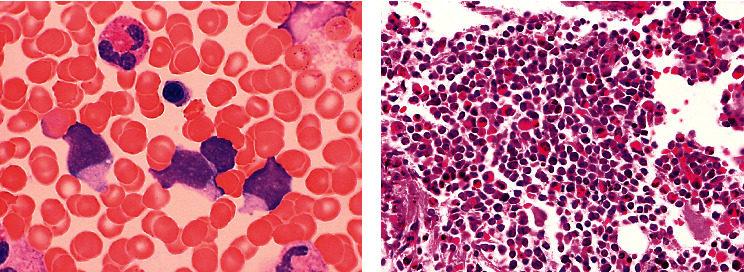
Bone marrow aspirate (BMA) and bone marrow biopsy (BMB): hematoxylin and eosin; 400x: (a) several agranular blasts and (b) numerous eosinophils and immature myeloid precursors.

Bone marrow aspirate and biopsy showed acute myeloid leukemia, minimally differentiated, with prominent eosinophilia and 28% blasts (Figure 2). Flow cytometry on the aspirate specimen revealed a prominent and discrete low side scatter, dim CD45 blast gate comprising approximately 30% of total events, expressing CD34 (partial, 50%), CD33, CD38, HLA-DR, CD7 (bright), CD123, and CD71, while negative for cytoplasmic TdT, myeloperoxidase (MPO), and all other lymphoid and monocytic markers (25 additional antigens tested). Bone marrow core biopsy was hypercellular (95%) with abundant immature mononuclear blast forms and eosinophils present. Immunohistochemistry was negative for CD34, CD117, MPO, TdT, and PAX5.

Chromosome analysis and fluorescence in situ hybridization (FISH) analysis were performed using standard cytogenetic methods by the Cytogenetics Laboratory at the University of Rochester Medical Center. Twenty metaphase spreads were analyzed, and no clonal aberrations were detected. FISH analysis was performed on 200 interphase nuclei and revealed a normal pattern for each of the following probes: *PDGFRA* (4q12), *PDGFRB, BCR/ABL*1, *RUNX*1*T*1*/RUNX*1, *KMT*2*A*, and *CBFB.* Additional FISH analysis performed at a reference laboratory found no evidence of JAK2 and FGFR1 rearrangements. Taken together, cytogenetic analysis did not detect any recurrent gene rearrangements associated with eosinophilic myeloid neoplasms.

Mutation analysis was performed using next-generation sequencing on the Illumina (San Diego CA) MiSeqDx after library preparation using the Illumina TruSight Myeloid Sequencing Panel. Data analysis was performed using the Illumina MiSeq Reporter and VariantStudio programs, as well as custom software. Analysis revealed a pathogenic mutation in plant homeodomain finger 6 (*PFH*6) with VAF of 13%. The *PHF*6 mutation c.684_705delinsCCC causes a shift in the reading frame and premature termination of translation (p.His229ProfsTer44), thus deemed pathogenic as a chain-terminating mutation. Additionally, a mutation was found in ZRSR2 with VAF 73%. The ZRSR2 mutation c.1332_1343dup is an in-frame duplication of 4 amino acids (p.Ser445_Arg448dup). It has been reported in the gnomAD exomes database at about 0.2% and is likely not pathogenic.

Therapy was initiated with intravenous methylprednisolone 125 mg daily for 3 days followed by oral prednisone 1 mg/kg for 4 days. His symptoms improved, and his peripheral blood blast count was reduced to 1%, while the absolute peripheral blood eosinophil count was reduced to 10.6 × 10^9^/L. This was followed by standard chemotherapy for AML with cytarabine (100 mg/m^2^ by intravenous continuous infusion on days 1–7) and idarubicin (12 mg/m^2^ intravenously on days 1–3). His day 14 bone marrow biopsy was hypocellular and consistent with chemotherapy-induced ablation, with no morphologic evidence of leukemia. A postinduction biopsy on day 39 was consistent with complete remission with complete hematologic recovery. He had one cycle of consolidative high-dose cytarabine followed by matched sibling donor allogeneic stem cell transplantation. He is in a sustained complete remission at the time of this report, with no evidence of relapse at 6 months after initial diagnosis.

## 3. Discussion

The majority of myeloid neoplasms that present with prominent eosinophilia are associated with rearrangements in the platelet-derived growth factor receptor *α* (*PDGFRA*) or *β* (*PDGFRB*), and fibroblast growth factor receptor 1 (*FGFR*1), which lead to constitutively active tyrosine kinase fusion genes, and often manifest as MPNs [2]. It is important to assess whether an underlying *PDGFR* rearrangement is present because even upon transition to a blast phase disease, these can remain sensitive to tyrosine kinase inhibition [3, 4]. Bone marrow eosinophilia is a feature of core-binding factor AML with t(8; 21) (q22; q22.1) or inv(16) (p13.1q22), which is associated with a favorable prognosis due to a high degree of chemosensitivity [5]. Marrow eosinophilia has also been described in rare forms of myelomonocytic leukemia. ETV6 fusions with different tyrosine kinase proteins including *ABL*1, *FLT*3, *JAK*2, and *FGFR*3 are associated with leukemogenesis and hypereosinophilia [6]. In these cases, prognosis has largely been determined by the fusion partner and responsiveness to different targeted therapies.

In this report, we describe a patient presenting with acute myeloid leukemia with minimal differentiation and prominent eosinophilia, with a pathogenic mutation in *PHF*6 at low variant allele frequency. *PHF*6 is a tumor suppressor gene with its locus on the X-chromosome (Xq26.2). It is recurrently mutated in T-cell acute lymphoblastic lymphoma (ALL) and occurs in about 3% of all AML cases, much more commonly in males than females [7]. Little is known about the exact mechanisms by which *PHF*6 mutations may contribute to the pathogenesis of AML or eosinophilia. The majority of these mutations are loss of function mutations, as it was in our case. They are commonly seen without evidence of cytogenetic abnormalities but with additional mutations in *ASXL*1, *RUNX*1, *TET*2, and *DNMT*3*A*. In this case, the patient had a normal karyotype analysis and a mutation in an additional gene located on the X-chromosome, *ZRSR*2, which has been detected in 1–3% of AML cases and has no known prognostic or therapeutic implications [8, 9]. To our knowledge, this is the first reported case of *PHF*6*-*mutated acute myeloid leukemia that presented with prominent eosinophilia, with clinical and radiographic evidence of associated organ involvement. Previous reports have been called either acute eosinophilic leukemia (AEL) or acute myeloid leukemia with eosinophilia [10, 11]. Eosinophil dysplasia has been noted in both benign and neoplastic disorders but may be helpful in the differential diagnosis of potential myeloid neoplasms [12]. We prefer the term AEL as being consistent with other AML subtypes.

There are limited data to guide the management of these patients due to the rarity of the condition. Our report highlights that, in cases of AEL, it is important to initiate the standard cytogenetic and molecular analysis that is performed in new cases of AML, in addition to evaluation for aberrations that are associated with eosinophilia. In the absence of targetable mutations or chromosomal rearrangements that may inform management, one reasonable approach is the use of high-dose glucocorticoids for symptomatic hypereosinophilia with organ involvement, as well as standard induction chemotherapy followed by allogeneic stem cell transplant for the treatment of AEL. Additional studies are required for better characterization of these patients and determination of the optimal treatment strategies to result in long-term remission.
